# Image quality and subject experience of quiet T1-weighted 7-T brain imaging using a silent gradient coil

**DOI:** 10.1186/s41747-022-00293-x

**Published:** 2022-08-31

**Authors:** Sarah M. Jacobs, Edwin Versteeg, Anja G. van der Kolk, Leonie N. C. Visser, Ícaro A. F. Oliveira, Emiel van Maren, Dennis W. J. Klomp, Jeroen C. W. Siero

**Affiliations:** 1 Department of Radiology and Nuclear Medicine, University Medical Center Utrecht, Utrecht, the Netherlands; 2 Department of Medical Imaging, Radboud University Medical Center, Nijmegen, the Netherlands; 3 Department of Neurology, Alzheimer Center Amsterdam, Amsterdam Neuroscience, Vrije Universiteit Amsterdam, Amsterdam UMC, Amsterdam, the Netherlands; 4 Division of Clinical Geriatrics, Department of Neurobiology, Care Sciences and Society, Center for Alzheimer Research, Karolinska Institute, Stockholm, Sweden; 5 Spinoza Centre for Neuroimaging Amsterdam, Amsterdam, the Netherlands; 6 Experimental and Applied Psychology, VU University, Amsterdam, the Netherlands

**Keywords:** Acoustics, Healthy volunteers, Magnetic resonance imaging, Neuroimaging, Noise

## Abstract

**Objectives:**

Acoustic noise in magnetic resonance imaging (MRI) negatively impacts patients. We assessed a silent gradient coil switched at 20 kHz combined with a T_1_-weighted magnetisation prepared rapid gradient-echo (MPRAGE) sequence at 7 T.

**Methods:**

Five healthy subjects (21–29 years; three females) without previous 7-T MRI experience underwent both a quiet MPRAGE (Q-MPRAGE) and conventional MPRAGE (C-MPRAGE) sequence twice. Image quality was assessed quantitatively, and qualitatively by two neuroradiologists. Sound level was measured objectively and rated subjectively on a 0 to 10 scale by all subjects immediately following each sequence and after the whole examination (delayed). All subjects also reported comfort level, overall experience and willingness to undergo the sequence again.

**Results:**

Compared to C-MPRAGE, Q-MPRAGE showed higher signal-to-noise ratio (10%; *p* = 0.012) and lower contrast-to-noise ratio (20%; *p* < 0.001) as well as acceptable to good image quality. Q-MPRAGE produced 27 dB lower sound level (76 *versus* 103 dB). Subjects reported lower sound level for Q-MPRAGE both immediate (4.4 ± 1.4 *versus* 6.4 ± 1.3; *p* = 0.007) and delayed (4.6 ± 1.4 *versus* 6.3 ± 1.3; *p* = 0.005), while they rated comfort level (7.4 ± 1.0 *versus* 6.1 ± 1.7; *p* = 0.016) and overall experience (7.6 ± 1.0 *versus* 6.0 ± 0.9; *p* = 0.005) higher. Willingness to undergo the sequence again was also higher, however not significantly (8.1 ± 1.0 *versus* 7.2 ± 1.3; *p* = 0.066).

**Conclusion:**

Q-MPRAGE using a silent gradient coil reduced sound level by 27 dB compared to C-MPRAGE at 7 T while featuring acceptable-to-good image quality and a quieter and more pleasant subject experience.

## Key points

- The silent gradient coil allowed to obtain images of acceptable-to-good quality while reducing the sound level of T_1_-weighted magnetisation prepared rapid gradient-echo (MPRAGE) brain imaging at 7 T by 27 dB.
- Healthy subjects experienced this quiet T_1_-weighted MPRAGE brain imaging as quieter and more pleasant.
- The silent gradient coil enabled fast and quiet T_1_-weighted brain imaging at 7 T, showing a promising potential for a wide variety of clinical sequences.

## Background

Despite the many advantages that magnetic resonance imaging (MRI) offers, one of its main disadvantages is the loud acoustic noise it generates [1, 2]. Acoustic noise can hinder adequate communication during the MRI examination and cause anxiety and transient hearing loss. This goes as far as a temporary hearing impairment in 43% of patients regardless of hearing protection, mild anxiety as experienced by 35% of patients and even severe panic and/or claustrophobia in 5–10% [3, 4]. Moreover, even a direct correlation between the acoustic noise level and claustrophobia has been demonstrated [5].

Patient discomfort can lead to motion artifacts or even unsuccessful completion of the MRI examination, resulting in potentially impaired diagnostics. In addition, there are several specific patient groups that would benefit from a quieter examination. Neonates and children often need to be sedated to successfully undergo an MRI examination due to the acoustic noise [6–8], whereas elderly or people with a psychiatric disorder can be triggered by the acoustic noise leading to sudden movement and reaction [9].

As the acoustic noise during an MRI examination is proportional to the amount of gradient switching, sound levels can be lowered by reducing gradient switching. State-of-the-art sound reduction sequences like pointwise encoding time reduction with radial acquisition, PETRA, and rotating ultrafast imaging sequence, RUFIS, are, therefore, based on zero echo time (ZTE) imaging. ZTE imaging involves almost no gradient switching and is virtually silent [10, 11]. However, applying these ZTE sequences to all image contrasts is challenging as they use a short echo time (TE) radial acquisition, which features longer acquisition times than conventional sequences to limit artifacts [12, 13]. Recently, an alternative approach to reduce sound has been proposed, which increases the gradient switching frequency beyond the hearing threshold using a silent gradient coil [14]. This coil can be combined with a reduced slew rate and amplitude on the whole-body gradients to further reduce sound involving only minimal modifications for conventional sequences to preserve image contrast.

In this study, we used a silent gradient coil at 7 T that is switched at the inaudible frequency of 20 kHz with a silent readout module implemented in a *magnetisation prepared rapid gradient-echo* (MPRAGE) sequence [15]. The MPRAGE sequence was chosen because of its known high acoustic noise levels and the important role of T_1_-weighted imaging in brain MRI diagnostics. We assessed image quality and subject experience in healthy subjects and measured the objective sound level of the implemented quiet MPRAGE sequence compared to a conventional MPRAGE sequence.

## Methods

### Study population

To achieve the most MRI ‘naïve’ experience, five healthy subjects (aged 21–29 years; three females) with no previous 7-T MRI experience were included. This study was approved by the local ethics committee and in compliance with national legislation and the Declaration of Helsinki; all subjects provided written informed consent.

### Experimental setup

Subjects were imaged with both the quiet and conventional sequence during the same MRI examination. MRI examinations were performed with the silent gradient coil (Futura Composites, Heerhugowaard, the Netherlands) positioned in a 7-T MRI scanner (Achieva, Philips, Best, the Netherlands). The silent gradient coil was fitted with a 32-channel receive array (Nova Medical, Wilmington, MA, USA).

**Fig. 1:**
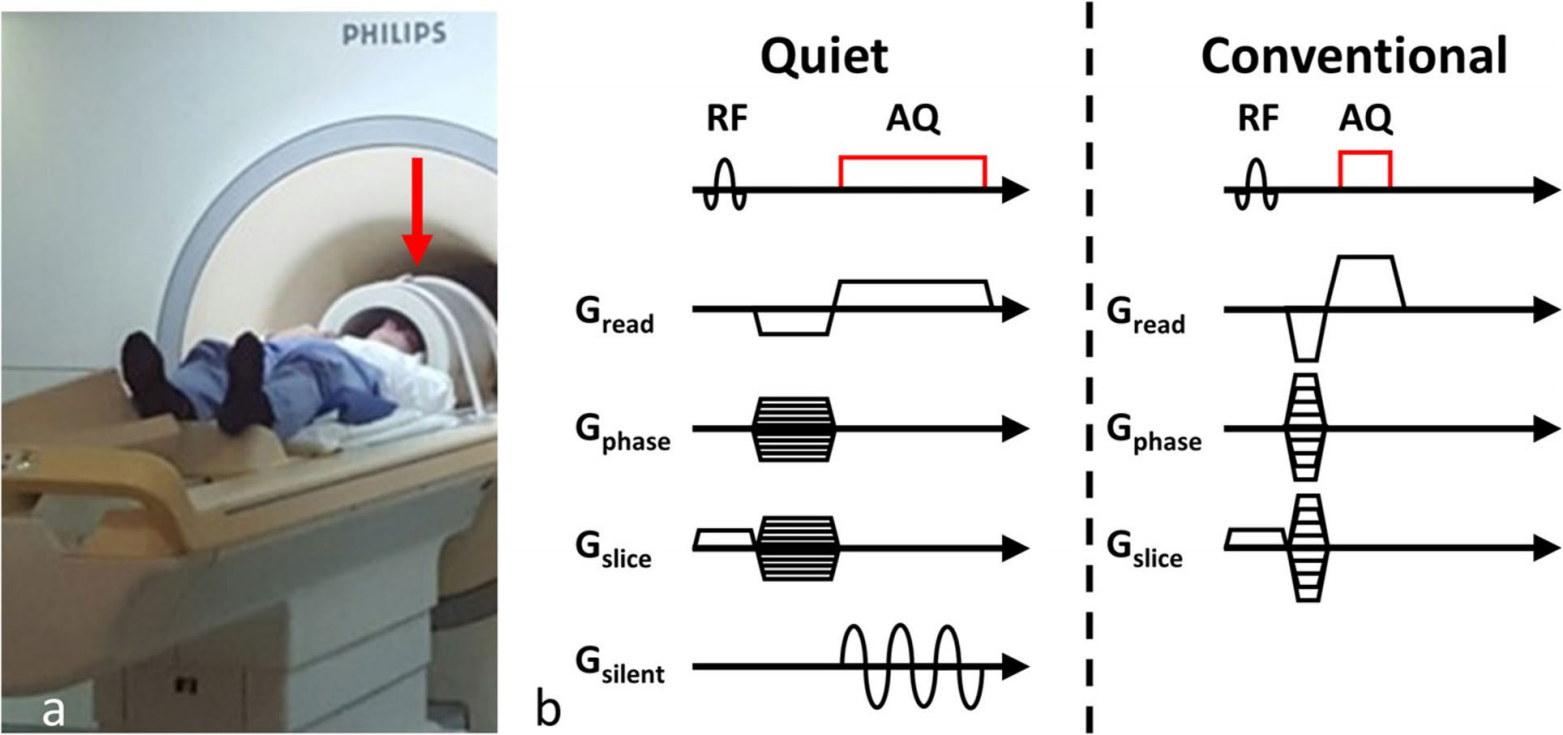
**a** The silent gradient coil used in this work (indicated by the red arrow). **b** Sequence diagrams of the readout of the quiet and conventional MPRAGE. The quiet MPRAGE features lower slew rates and amplitudes to limit sound from the audible gradients and incorporates an extra silent gradient during the readout to improve imaging efficiency. *MPRAGE* Magnetisation prepared rapid gradient-echo

The silent gradient coil consists of a resonant single-coil head insert gradient coil combined with an audio amplifier that enables ~20 kHz switching with adequate power [14]. This gradient insert operates in the *z*-direction (feet-head), features an integrated radiofrequency transmit coil and can be switched off between examinations (Fig. 1a). The silent gradient coil can in principle operate at a maximum gradient amplitude and slew rate of 40 mT/m and 5,200 T/m/s. In comparison, a conventional whole-body gradient system operates at a gradient amplitude of around 40 mT/m and is limited by peripheral nerve stimulation to a maximum slew rate of 200 T/m/s. However, the small size of the silent gradient coil produced no noticeable peripheral nerve stimulation despite the order of magnitude higher slew rate. In this work, the silent gradient coil was driven at a gradient amplitude of 28.6 mT/m to limit heating of the audio amplifier due to the high duty cycle of the MPRAGE sequence.

### Imaging protocol

Both sequences featured a field of view of 240 × 240 × 172 mm^3^ and 1.0 mm isotropic resolution. The quiet sequence used optimised imaging parameters and a gradient mode to reduce sound, while the conventional sequence used standard clinical parameters and gradient mode. The sequences differed primarily in their TE and repetition time (TR) which were 8.9 ms and 17.6 ms for the quiet sequence and 1.9 ms and 4.2 ms for the conventional sequence, respectively. The acquisition time was 2:44 min:s for the quiet sequence and 2:24 min:s for the conventional sequence. Other imaging parameters can be found in Table 1. Images were reconstructed offline in MATLAB (MathWorks, Natick, MA, USA) using an iterative sensitivity encoding, SENSE, reconstruction. For the quiet sequence, the spatiotemporal behaviour of the oscillating gradient field was characterised using a field camera (Skope, Zürich, Switzerland) and used as an input for the reconstruction.

**Table 1:** Imaging parameters of the quiet and conventional sequence

	Quiet	Conventional
**Field of view**	240 × 240 × 172 mm^3^	240 × 240 × 172 mm^3^
**Voxel size**	1.0 × 1.0 × 1.0 mm^3^	1.0 × 1.0 × 1.0 mm^3^
**Echo time**	8.9 ms	1.9 ms
**Repetition time**	17.6 ms	4.2 ms
**Flip angle**	13°	7°
**Shot interval**	3,000 ms	3,000 ms
**Inversion time**	1,000 ms	1,000 ms
**Acceleration**	Not used	2 × 1.4 (phase × slice)
**Acquisition time**	2:44 min:s	2:24 min:s

The quiet sequence featured a silent readout module consisting of a silent 20 kHz readout gradient that was applied simultaneously with the whole-body encoding gradients of a conventional MPRAGE sequence. The acoustic noise is reduced by using a reduced slew rate and gradient amplitude for the whole-body encoding gradients, which generally leads to longer repetition time and acquisition time. However, the silent readout provides extra spatial encoding during each readout without introducing extra acoustic noise, leading to fewer encoding steps to form an image and therefore reducing the total acquisition time. In summary, this approach reduces the acoustic noise while minimally affecting the acquisition time. The silent readout module was combined with a controlled aliasing in parallel imaging, CAIPI, sampling pattern to limit image noise enhancement from variations in sample density introduced by the rapidly oscillating silent gradient [16]. A schematic representation of the sequence is displayed in Fig. 1b.

Importantly, the slow switching of the whole-body gradient still resulted in a longer TE and TR during the quiet sequence, which, when not addressed, results in suboptimal grey-white matter contrast and cerebrospinal fluid (CSF) nulling. Therefore, we performed signal simulations using extended phase graphs, EPG, which allowed us to simulate the grey-white matter contrast and CSF nulling for a range of TEs, TRs, and flip angles. The quiet sequence was simulated for a range of flip angles between 1 and 90°. The flip angle that generated the contrast that most closely matched the contrast in the conventional sequence was then chosen (assuming no radiofrequency inhomogeneities).

The inversion pulse determines the nulling of the CSF signal and grey-white matter contrast. In particular, the spatial homogeneity of the transmit radiofrequency field (B_1_) strongly influences the effectiveness of the inversion pulse. At higher magnetic field strengths (> 7 T), the B_1_ field becomes more inhomogeneous, resulting in a spatially varying image contrast. To ensure a more homogenous image contrast, we have implemented a time-resampled frequency-offset corrected inversion, TR-FOCI, inversion pulse [17], which is less sensitive to B_1_ field inhomogeneities than conventional inversion pulses. This inversion pulse was used for both the quiet and conventional sequence.

### Objective sound level measurements

The sound level during the quiet and conventional sequence was measured using an MRI safe condenser microphone (ECM8000, Behringer, Willich, Germany) connected to a computer, which recorded the sound directly using MATLAB. A 94 dB noise source (sound level calibrator type 4231, Bruel & Kjaer, Nærum, Denmark) was used to calibrate this microphone. During the sound measurements, the microphone was placed in the gradient insert without a subject being present and at a position that mimicked the position of the ears during the examination. The measurement data was processed in MATLAB, and exponential filtering and A-weighting were applied to correspond to the fast response setting and output of a sound level metre [18].

### Quantitative image assessment

For each subject, the images were skull-stripped using optiBET [19] allowing the registration of the quiet sequence images to the images of the conventional sequence using a rigid-body registration (FLIRT FSL toolbox) [20]. The grey-white matter contrast of the sequences was quantified using the signal-to-noise ratio (SNR), contrast-to-noise ratio (CNR) and tissue signal histograms. The grey and white matter were segmented from the skull-stripped images using the FAST automated segmentation tool from the FSL toolbox [21]. Importantly, bias field-corrected images were used to remove signal variations due to inhomogeneous B_1_. The segmentation output was analysed in MATLAB (MathWorks, Natick, MA, USA), and the SNR and CNR were calculated using Eqs. 3 and 4 from Oliveira et al. [22]:

1 $$SNR=\frac{\mu_{foreground}}{\sigma_{foreground}}\sqrt{n/\left(n-1\right)}$$

2 $$CNR=\frac{abs\left({\mu}_{white- matter}-{\mu}_{grey- matter}\right)}{\sqrt{\sigma_{white- matter}^2+{\sigma}_{grey- matter}^2}}$$

Here, the SNR was determined by calculating the ratio of the average signal μ_foreground_ and standard deviation (SD) *σ*_foreground_ in the combined grey and white matter, which was then scaled to the number of voxels (n) to allow for comparison between scans. The CNR was determined by calculating the absolute difference between the average signal in the grey (*μ*_grey-matter_) and white matter (*μ*_grey-matter_). The noise was estimated by combining the SDs in the grey (*σ*_grey-matter_) and white matter (*σ*_grey-matter_).

### Qualitative image assessment

Blind assessment of the registered skull-stripped images of both the quiet and the conventional sequences was performed by two neuroradiologists to determine the image quality: one with eleven years of experience in 7-T neuroimaging and a neuroradiology fellow with three years of neuroimaging experience. Overall image quality, visibility of anatomical details, grey-white matter contrast and delineation of vascular structures were scored using a 5-point Likert scale from 1 (very poor) to 5 (excellent). Visibility of anatomical details and grey-white matter contrast were divided into the following subcategories, *i.e,* areas of the brain: frontal, temporal, parietal and occipital lobe, limbic system, and basal ganglia. Additionally, flow, susceptibility, bounce point and truncation artifacts, if present, were scored from 1 (severe) to 4 (mild). An average score per category was determined for the quiet and conventional sequence.

### Subject experience

Subjects were given adequate hearing protection, *i.e.,* earplugs combined with earmuffs. Each subject underwent both the quiet and the conventional sequence twice to determine consistency in reporting; the order of the sequences differed between subjects to rule out any order effects. Immediately after each sequence and after the whole MRI examination (delayed), subjects were asked to rate the sound level of each sequence on a scale from 0 to 10, with 0 being absolutely silent and 10 being the loudest sound they could imagine. In addition, after the whole MRI examination, subjects completed a questionnaire in which they rated their level of comfort, overall experience and willingness to undergo the sequence again on a scale from 0 to 10. For level of comfort, 0 meant being extremely uncomfortable and 10 the most comfortable they could imagine; for overall experience, 0 meant not being satisfied at all and 10 extremely satisfied, and for willingness to undergo the sequence again, 0 meant being absolutely not willing and 10 very much willing to undergo this MRI sequence again in the future.

### Statistical analysis

For the quantitative image assessment, a Wilcoxon signed-rank test was used to assess the differences in SNR and CNR with a significance level of *p* < 0.05. A Cohen’s κ was calculated to determine the interobserver agreement for the qualitative image assessment scores for both the quiet and the conventional sequence.

Since all subjects underwent each sequence twice, differences in ratings between the first and second time were assessed first, after which an average rating per category was calculated. For each of the experience measures, differences in the experience ratings of both the quiet and the conventional sequence were assessed using Wilcoxon signed-rank tests with a significance level of *p* < 0.05.

## Results

### Sound level measurements

The peak sound level in the quiet sequence was measured to be 76 dB(A), which was 27 dB lower than the peak sound level measured during the conventional sequence which was 103 dB(A). The main source of the residual sound of the quiet sequence originated from the slowly switching whole-body gradients resulting in a low-frequency humming sound during the readout.

### Quantitative image assessment

**Fig. 2:**
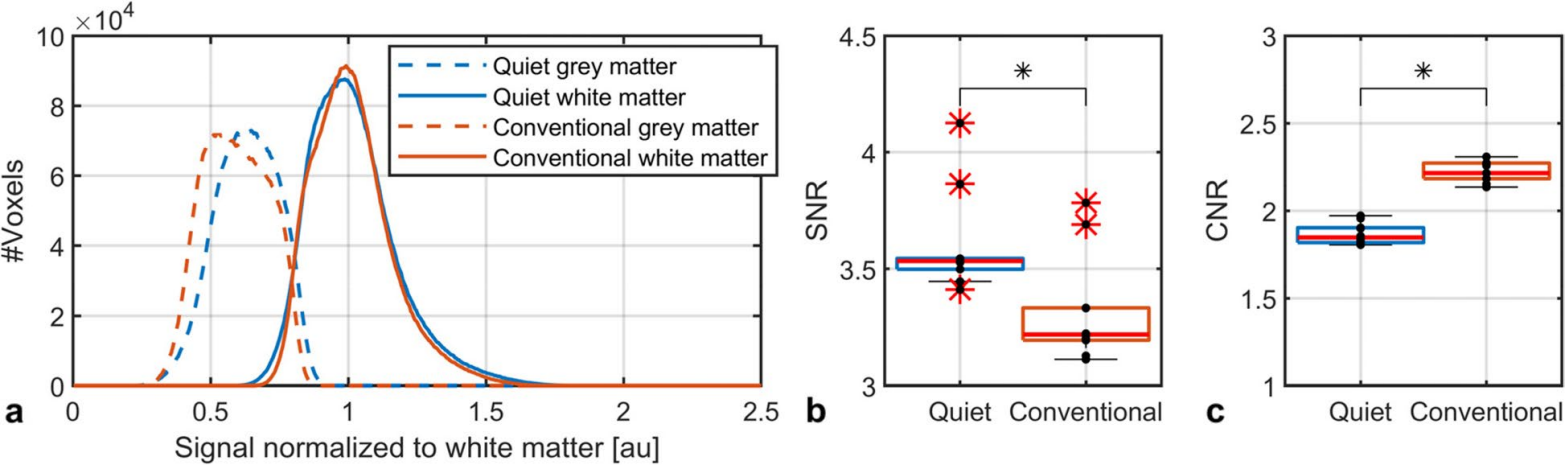
Results from quantitative image assessment. **a** Histogram of the normalised signal intensities of grey and white matter for the quiet and conventional sequence for all subjects. **b** Box plot of the signal-to-noise ratio in each subject. **c** Box plot of the contrast-to-noise ratio for all subjects. Asterisk indicates statistical significance (*p* < 0.05; Wilcoxon signed-rank test)

The histograms in Fig. 2a show the signal intensity distribution for grey and white matter in the whole brain. The white matter signal distributions were found to be similar for both sequences, while on average, the grey matter signal was found to be higher (6.7%). This higher average signal yielded a 10% (SD 3.6%) higher SNR in the quiet sequence compared to the conventional one, which was found to be significant (*p* = 0.002; Fig. 2b). However, consequently, the signal intensity of grey and white matter was more similar in the quiet sequence, resulting in a larger overlap of the grey and white matter signal intensity distributions and a 20% (SD 1.4%) lower CNR compared to the conventional sequence, which was found to be significant (*p* < 0.002; Fig. 2c).

### Qualitative image assessment

**Fig. 3:**
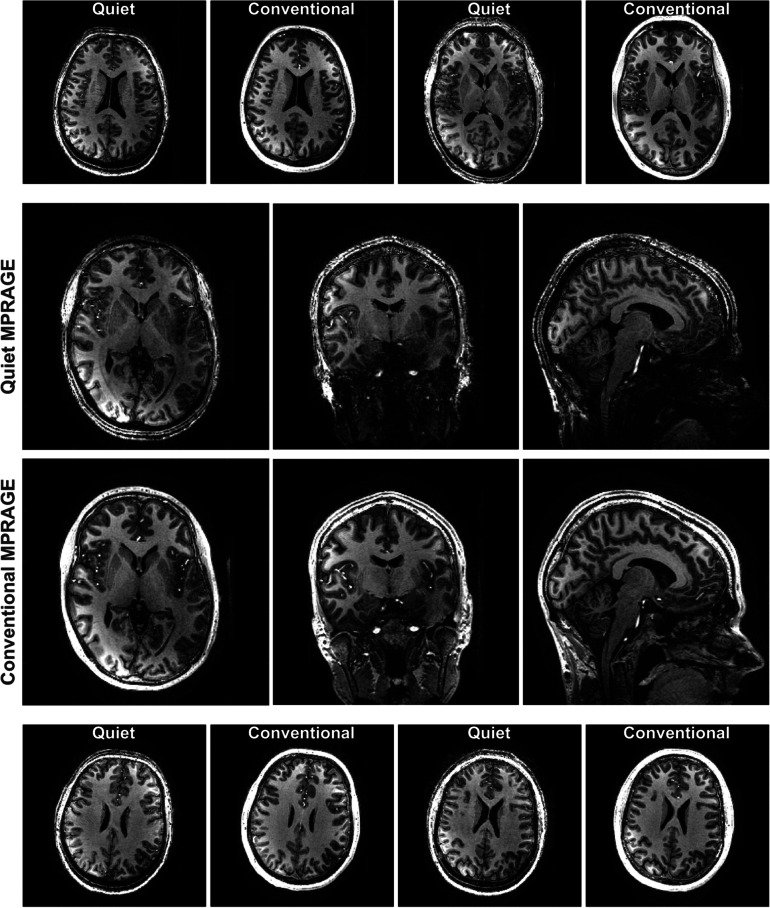
Middle cross-sections of both sequences for a representative subject; top and bottom, axial slices of the other four subjects

Average scores of all categories indicated similar image quality and artifacts between the quiet and the conventional sequence, with only 0–1-point difference (Fig. 3; Table 2). The image quality of the quiet sequence was deemed good or acceptable for all categories, with mild to moderate artifacts, except for the anatomical details in the temporal lobe. Observers pointed out lower image quality of the left compared to the right side of the brain in both sequences, leading them to give a lower score than when they had scored left and right separately. Interobserver agreement for both sequences was fair for both the quiet and conventional sequences (Cohen’s κ 0.40 and 0.38, respectively), apart from the artifacts where we chose to rely on the 7-T experienced radiologist.

**Table 2:** Average image scores of the two observers for both sequences on visibility of anatomical details and grey-white matter contrast and delineation of vascular structures  using a 5-point Likert scale: 1 very poor, 2 poor, 3 acceptable, 4 good, and 5 excellent

Criteria		Quiet	Conventional
Overall image quality		3	4
Anatomical details	Frontal lobe	3	4
	Temporal lobe	2	3
	Parietal lobe	4	4
	Occipital lobe	3	3
	Limbic system	3	3
	Basal ganglia	3	4
Grey-white matter contrast	Frontal lobe	4	4
	Temporal lobe	3	4
	Parietal lobe	4	4
	Occipital lobe	3	4
	Limbic system	3	4
	Basal ganglia	4	4
Vascular structures		3	4
Artifacts*	Flow	2	2
	Susceptibility	2	3
	Bounce point	2	2
	Truncation	3	3

*Average scores of one 7-T experienced neuroradiologist. Likert scale for image artifacts if present: 1 severe, 2 obvious, 3 moderate, 4 mild

### Subject experience

**Fig. 4:**
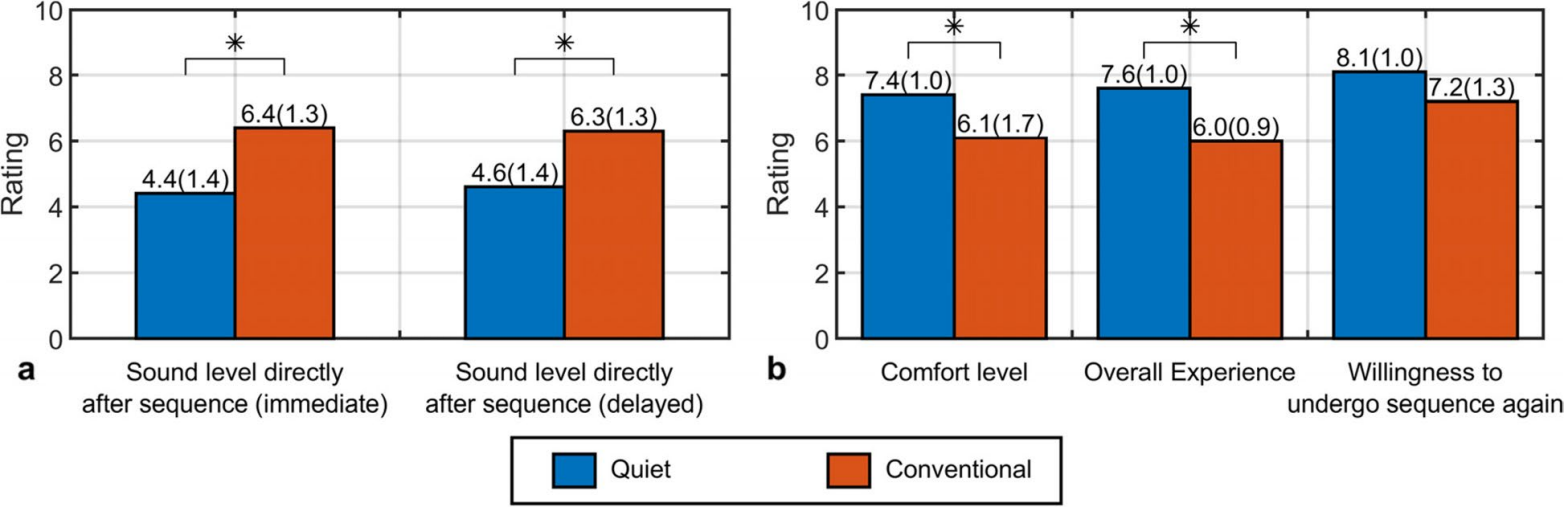
**a** Means (SD) of reported sound level ratings immediately after the sequence and after the whole examination (delayed) for the quiet compared to the conventional sequence. **b** Means (SD) of comfort level, overall experience and willingness to undergo sequence again ratings for the quiet compared to the conventional sequence. Asterisk indicates statistical significance (*p* < 0.05; Wilcoxon signed-rank test). *SD* Standard deviation

Mean differences between all ratings of the first and second sequence of the quiet and conventional sequence were minimal: 0.6 (SD 0.9) and 0.3 (SD 0.9) points, respectively. All subjects reported a substantially and significantly lower sound level of the quiet sequence, both immediate (4.4, SD 1.4; *p* = 0.007) and delayed (4.6, SD 1.4; *p* = 0.005) and rated comfort level (7.4, SD 1.0; *p* = 0.016) and overall experience (7.6, SD 1.0; *p* = 0.005) of the quiet sequence significantly higher (Fig. 4). Willingness to undergo the quiet sequence again was also higher (8.1, SD 1.0; *p* = 0.066), however not significant. An interesting remark from two subjects was that the type of sound of the quiet sequence was more pleasant to listen to.

## Discussion

This pilot study shows that our silent gradient coil with silent readout module incorporated into a T_1_-weighted MPRAGE sequence at 7 T delivers images of acceptable to good quality and is perceived as quieter and more pleasant by our subjects than the conventional MPRAGE. The unique features of our study are that it employs a dedicated silent encoding coil and thorough assessment of subject experience.

Three other studies regarding quiet anatomical brain imaging have been conducted at 7 T, and only two other studies have tested a quiet MPRAGE, all using a ZTE method [12, 23–26]. Compared to our method, these ZTE methods featured a lower sound level (50–60 dB *versus* 76 dB), albeit with approximately two times longer acquisition times due to a lack of implemented image acceleration schemes. To achieve a similar reduction in sound level using our method, the amplitude of the silent gradient would need to increase, as this could compensate for slower gradient switching, yet this requires improved gradient amplifier hardware.

Our subjects were blinded to the sequence, but not blinded to sound as we wanted them to focus specifically on the sound level they experienced. This is a limitation of our study design, as this could have led to information bias. We addressed blinding the subject to sequence type by repeating the sequences in a random and different order per subject. Additionally, we asked the questions about level of comfort, overall experience and willingness to undergo the sequence again before the delayed sound level question.

Quantitative image assessment showed a 20% (SD 1.4%) lower grey-white matter contrast of the quiet sequence compared to the conventional one. This primarily originated from the increased sensitivity of the quiet sequence to B_1_ field inhomogeneities and susceptibility (longer TE), both limitations of our sequence, translating in signal distortion and signal loss. Fortunately, the difference observed in contrast and CNR was not as apparent in the qualitative image assessment, as most categories had similar average scores for both the quiet and conventional sequence with a maximum difference of one point. The only area of concern was the temporal lobe, where both observers based their score on the left side of the brain, leading to an overall lower score. This left-right difference in the temporal lobe was caused by a lower B_1_ in the left temporal lobe, which might be improved using dielectric pads or an improved transmit coil design [27].

A final limitation of our study was that subject experience was only obtained from healthy subjects. As a next step, subject experience will be assessed in small cohorts of patients that could actually benefit from a quieter MRI examination, such as children, elderly and people with a psychiatric disorder. In addition, other sequences like fluid-attenuated inversion recovery (FLAIR) and susceptibility-weighted imaging (SWI) will also benefit from less acoustic noise. The application of the silent gradient coil to other sequences like FLAIR and SWI should in principle be more straightforward than for MPRAGE investigated here as very minimal changes in sequence design are necessary. However, the influence of longer TE and TR on the desired image contrast should be investigated for any new applications featuring a short TR (*e.g,* FLAIR). In this work, our silent gradient coil was applied at 7 T as this is the field strength with the highest sound level and has greater SNR. Translation to lower field strengths (1.5 or 3 T) is possible and would yield even lower sound levels due to the scaling of acoustic noise with field strength [28].

In conclusion, a quiet T_1_-weighted MPRAGE sequence at 7 T using a silent gradient coil reduces sound by 27 dB compared to a conventional MPRAGE sequence while featuring acceptable to good image quality of the brain and a quieter and more pleasant subject experience. A silent gradient coil provides a way for fast and quiet brain imaging with the promising potential to greatly improve patient comfort for a wide variety of clinical sequences.

## Data Availability

The datasets used and/or analysed during the current study are available from the corresponding author on reasonable request.

## Abbreviations

CNR: Contrast-to-noise ratio
CSF: Cerebrospinal fluid
MPRAGE: Magnetisation-prepared rapid gradient echo
MRI: Magnetic resonance imaging
SD: Standard deviation
SNR: Signal-to-noise ratio
TE: Echo time
TR: Repetition time
ZTE: Zero echo time

## References

[1] McJury MJ (2021). Acoustic noise and magnetic resonance imaging: a narrative/descriptive review. J Magn Reson Imaging.

[2] McNulty JP, McNulty S (2009). Acoustic noise in magnetic resonance imaging: an ongoing issue. Radiography.

[3] Brummett RE, Talbot JM, Charuhas P (1988). Potential hearing loss resulting from MR imaging. Radiology.

[4] Phillips S, Deary IJ (1995). Interventions to alleviate patient anxiety during magnetic resonance imaging: a review. Radiography.

[5] Dewey M, Schink T, Dewey CF (2007). Claustrophobia during magnetic resonance imaging: cohort study in over 55,000 patients. J Magn Reson Imaging.

[6] Moelker A, Maas RAJJ, Pattynama PMT (2004). Verbal communication in MR environments: effect of MR system acoustic noise on speech understanding. Radiology.

[7] McJury M, Shellock FG (2000). Auditory noise associated with MR procedures: a review. J Magn Reson Imaging.

[8] Edwards AD, Arthurs OJ (2011). Paediatric MRI under sedation: is it necessary? What is the evidence for the alternatives?. Pediatr Radiol.

[9] Shellock FG (1994). Magnetic resonance: bioeffects, safety, and patient management.

[10] Grodzki DM, Jakob PM, Heismann B (2012). Ultrashort echo time imaging using pointwise encoding time reduction with radial acquisition (PETRA). Magn Reson Med.

[11] Madio DP, Lowe IJ (1995). Ultra-fast imaging using low flip angles and fids. Magn Reson Med.

[12] Weiger M, Brunner DO, Dietrich BE, Müller CF, Pruessmann KP (2013). ZTE imaging in humans. Magn Reson Med.

[13] Ljungberg E, Damestani NL, Wood TC (2021). Silent zero TE MR neuroimaging: current state-of-the-art and future directions. Prog Nucl Magn Reson Spectrosc.

[14] Versteeg E, Klomp DWJ, Siero JCW (2022). A silent gradient axis for soundless spatial encoding to enable fast and quiet brain imaging. Magn Reson Med.

[15] Mugler JP, Brookeman JR (1990). Three-dimensional magnetization-prepared rapid gradient-echo imaging (3D MP RAGE). Magn Reson Med.

[16] Breuer FA, Blaimer M, Mueller MF (2006). Controlled aliasing in volumetric parallel imaging (2D CAIPIRINHA). Magn Reson Med.

[17] Hurley AC, Al-Radaideh A, Bai L (2010). Tailored RF pulse for magnetization inversion at ultrahigh field. Magn Reson Med.

[18] Houser DS, Yost W, Burkard R, Finneran JJ, Reichmuth C, Mulsow J (2017). A review of the history, development and application of auditory weighting functions in humans and marine mammals. J Acoust Soc Am.

[19] Lutkenhoff ES, Rosenberg M, Chiang J (2014). Optimized brain extraction for pathological brains (optiBET). PLoS One.

[20] Jenkinson M, Beckmann CF, Behrens TEJ, Woolrich MW, Smith SM (2012). FSL. Neuroimage.

[21] Zhang Y, Brady M, Smith S (2001). Segmentation of brain MR images through a hidden Markov random field model and the expectation-maximization algorithm. IEEE Trans Med Imaging.

[22] Oliveira ÍAF, Roos T, Dumoulin SO, Siero JCW, van der Zwaag W (2021). Can 7T MPRAGE match MP2RAGE for gray-white matter contrast?. Neuroimage.

[23] Ida M, Wakayama T, Nielsen ML, Abe T, Grodzki DM (2015). Quiet T1-weighted imaging using PETRA: initial clinical evaluation in intracranial tumor patients. J Magn Reson Imaging.

[24] Aida N, Niwa T, Fujii Y et al (2016) Quiet T1-weighted pointwise encoding time reduction with radial acquisition for assessing myelination in the pediatric brain. AJNR Am Neuroradiol 37:1528–1534. 10.3174/ajnr.A4747 10.3174/ajnr.A4747 PMC7960287 27056422

[25] Beenakker J-WM, Wezel J, Groen J, Webb AG, Börnert P (2019). Silent volumetric multi-contrast 7 Tesla MRI of ocular tumors using zero echo time imaging. PLoS One.

[26] Costagli M, Symms MR, Angeli L (2016). Assessment of silent T1-weighted head imaging at 7 T. Eur Radiol.

[27] O’Brien KR, Magill AW, Delacoste J (2014). Dielectric pads and low-B1+ adiabatic pulses: complementary techniques to optimize structural T1w whole-brain MP2RAGE scans at 7 Tesla. J Magn Reson Imaging.

[28] Theysohn JM, Maderwald S, Kraff O, Moenninghoff C, Ladd ME, Ladd SC (2008) Subjective acceptance of 7 Tesla MRI for human imaging. MAGMA 21:63–72. 10.1007/s10334-007-0095-x 10.1007/s10334-007-0095-x 18064501

